# Differential item functioning of patient‐reported outcomes measures of anxiety and depression by severity of cognitive impairment

**DOI:** 10.1002/alz.089740

**Published:** 2025-01-09

**Authors:** Andrea Russell, Rebecca Lovett, Abigail Vogeley, Stephanie Batio, Lauren Opsasnick, Eileen Graham, Daniel Mroczek, Morgan Bonham, Julia Yoshino‐Benavente, Rachel O'Conor, Michael S Wolf

**Affiliations:** ^1^ Northwestern University, Chicago, IL USA; ^2^ Northwestern University Feinberg School of Medicine, Chicago, IL USA

## Abstract

**Introduction:**

Patient‐reported outcomes are increasingly being utilized in clinical settings to identify psychological symptoms and track fluctuations over time. Some clinicians and researchers have expressed concerns about the validity of symptom questionnaires cognitively impaired populations. We sought to determine if differential item functioning (DIF) is present based on cognitive impairment using the patient reported outcomes measurement information system (PROMIS) inventories of anxiety and depression.

**Methods:**

Data from a longitudinal aging cohort study in Chicago, IL. Mild impairment was defined as a z‐score between ‐1 and ‐1.49 and moderate/severe impairment was a z‐score of ≤‐1.5 on 2+ tests in one of 5 cognitive domains. PROMIS Anxiety and Depression measures were utsed. Due to sample size and response variation, the anxiety model examined DIF with participants categorized using three categories (none, mild, or moderate/severe cognitive impairment). The depression model used 2 categories (none/mild vs moderate/severe impairment). Analyses were conducted in R (*lordif package* with Monte Carlo simulations). Effect sizes for DIF were calculated by test item. Test characteristic curves (TCCs) examined measure performance by cognitive impairment.

**Results:**

Data was available for 396 participants, who were 71 years old, mostly female (n = 285) and about half were non‐Hispanic White (n = 214). Approximately 72% had no cognitive impairment, 14% had mild cognitive impairment and 13% had moderate/severe impairment. There was evidence of DIF in 4 of 7 anxiety items and 3 of 8 depression items. The direction of DIF varied, suggesting the response patterns of moderately/severely impaired participants resulted in psychometric overestimation of anxiety and underestimation of depression. Effect sizes of DIF were relatively small for anxiety (0.016‐0.034) and depression (0.014‐0.043) and TCC curves were similar by cognitive impairment category for both measures.

**Conclusion:**

There is psychometric measurement bias on PROMIS anxiety and depression measures by severity of cognitive impairment. Researchers and clinicians should be aware that cognitive impairment may impact responses when using assessments, however, this study showed this bias is small and results in negligible change when examining the measures overall. These findings support the conclusion that PROMIS Anxiety and Depression are reliable assessments when used among individuals with different levels of cognitive impairment.

